# Prevalence and Crucial Parameters in Diabesity-Related Liver Fibrosis: A Preliminary Study

**DOI:** 10.3390/jcm12247760

**Published:** 2023-12-18

**Authors:** Szymon Suwała, Aleksandra Białczyk, Kinga Koperska, Alicja Rajewska, Magdalena Krintus, Roman Junik

**Affiliations:** 1 Department of Endocrinology and Diabetology, Nicolaus Copernicus University, Collegium Medicum, 9 Sklodowskiej-Curie Street, 85-094 Bydgoszcz, Poland; junik@cm.umk.pl; 2 Evidence-Based Medicine Students Scientific Club of Department of Endocrinology and Diabetology, Nicolaus Copernicus University, Collegium Medicum, 9 Sklodowskiej-Curie Street, 85-094 Bydgoszcz, Poland; kontakt@aleksandrabialczyk.eu (A.B.); kingakoperska@gmail.com (K.K.); alicja.p.rajewska@gmail.com (A.R.); 3 Department of Laboratory Medicine, Nicolaus Copernicus University, Collegium Medicum, 9 Sklodowskiej-Curie Street, 85-094 Bydgoszcz, Poland; krintus@cm.umk.pl

**Keywords:** liver fibrosis, hepatofibrosis, diabetes, obesity, diabesity, diagnostic

## Abstract

Diabetes and obesity have been recognized as confirmed risk factors for the occurrence of liver fibrosis. Despite the long-standing acknowledgment of “diabesity”, the simultaneous existence of diabetes and obesity, scholarly literature has shown limited attention to this topic. The aim of this pilot study was to assess the prevalence of liver fibrosis among individuals with diabetes (specifically those who are obese) in order to identify the key factors associated with hepatofibrosis and determine the most important associations and differences between patients with and without liver fibrosis. The research included a total of 164 participants (48.17% had comorbid obesity). Liver elastography (Fibroscan) was performed on these individuals in addition to laboratory tests. Liver fibrosis was found in 34.76% of type 2 diabetes patients; male gender almost doubled the risk of hepatofibrosis (RR 1.81) and diabesity nearly tripled this risk (RR 2.81; however, in degree III of obesity, the risk was elevated to 3.65 times higher). Anisocytosis, thrombocytopenia, or elevated liver enzymes raised the incidence of liver fibrosis by 1.78 to 2.47 times. In these individuals, liver stiffness was negatively correlated with MCV, platelet count, and albumin concentration; GGTP activity and HbA1c percentage were positively correlated. The regression analysis results suggest that the concentration of albumin and the activity of GGTP are likely to have a substantial influence on the future management of liver fibrosis in patients with diabesity. The findings of this study can serve as the basis for subsequent investigations and actions focused on identifying potential therapeutic and diagnostic avenues.

## 1. Introduction

Obesity is a widespread global phenomenon that is closely linked to the prevalence of noncommunicable diseases, including hypertension, cardiovascular disorders, and diabetes. Type 2 diabetes mellitus (T2DM) is a significant health concern, with its anticipated prevalence expected to rise to 12.2% (783.2 million individuals) by the year 2045, compared to the current rate of 10.5% in 2021 [1]. The interaction between cardiovascular and metabolic diseases has received considerable attention within the medical and scientific communities. This is apparent in the acknowledgment of particular medical conditions, including metabolic syndrome; non-alcoholic fatty liver disease (NAFLD); and, more recently, metabolic-associated fatty liver disease (MAFLD).

According to estimates, 30% of the global population has at least one liver disease risk factor [2]. One of them is obesity, as Kechagias et al. found that a 10% increase in body weight owing to overeating and fast-food consumption increases hepatic fat accumulation by 2.5-fold in 4 weeks [3]. Furthermore, the deposition of triglycerides in the liver and the development of hepatic steatosis, along with the activation of pro-inflammatory M1 macrophages in adipose tissue and the subsequent secretion of cytokines like interleukin 6 or tumor necrosis factor (TNF)-α, play a role in the advancement of hepatic insulin resistance [4]. About 40% of NAFLD patients develop non-alcoholic steatohepatitis (NASH). This disorder can develop to liver fibrosis, which can be reversed but leads to irreversible cirrhosis [5,6]. Endotoxins originating from the gastrointestinal tract, specifically lipopolysaccharide, could exert a substantial influence on hepatic inflammation and the progression of chronic liver ailments. The mechanism through which endotoxin is transported from the intestinal lumen into the portal vein system is an essential process, as the absorbed endotoxin is rapidly cleared by the hepatic reticuloendothelial system, particularly the Kupffer cells [7,8]. Obesity and T2DM may change intestinal permeability, causing bacteria overgrowth and mucus layer damage. Bacterial translocation can release gastrointestinal endotoxin into the systemic circulation. Invasive infections and their byproducts increase liver lipid buildup and pro-inflammatory and fibrotic processes [9,10,11].

The mortality rates in patients diagnosed with NAFLD demonstrate a gradual increase corresponding to the degree of fibrosis. As a result, the severity of hepatic fibrosis is currently recognized as the primary prognostic factor for this specific group of individuals. In their study, Sanyal et al. found a significant association between all-cause mortality and fibrosis stage progression, with mortality rates of 0.32 to 0.89 per 100 person-years for stages F1-F2 to F3 and 1.76 for stage F4 [12,13,14].

Despite the well-established correlation between obesity and T2DM, both of which are key components of the metabolic syndrome, there has been little scientific research on the prevalence of liver fibrosis in people with both conditions. Given the health risks of hepatofibrosis and the global prevalence of T2DM and obesity, it is crucial to thoroughly investigate and analyze this correlation. Assessing the problem and finding clinical connections should be the priority. This will help create a predictive strategy and maybe preventive action.

The aim of this research was to assess the prevalence of liver fibrosis among individuals diagnosed with diabetes (with a particular focus on those who also have obesity, referred to as “diabesity”) and to investigate potential associations with different anthropometric and laboratory parameters, including blood morphology, aminotransferases, coagulation, carbohydrate and lipid metabolism parameters, and liver stiffness measured by an objective and validated method of transient elastography examination (Fibroscan), and finally to determine which of these parameters can play a crucial role in evaluating the risk of liver fibrosis. The authors classified this study as preliminary, as its primary objective is to lay the groundwork for subsequent, more targeted investigations in the future.

## 2. Materials and Methods

### 2.1. Participants

During the initial phase of participant recruitment for the study, a group of 220 individuals diagnosed with T2DM was selected (mainly sourced from the diabetology outpatient clinic: 109 male and 111 female individuals). The participants were given an overview of the study procedures, after which 39 individuals chose to discontinue their participation. The 181 patients remaining in stage II were reviewed for acute or chronic viral hepatitis, drug-induced liver injury, autoimmune hepatitis, Wilson’s disease, hemochromatosis, and a history of alcohol abuse (exclusion criteria); 14 patients were rejected. A total of 167 study participants underwent the collection of biological samples with the aim of identifying the existence of anti-HBs and anti-HCV antibodies; among these participants, it was found that 3 patients were newly diagnosed with viral hepatitis. In the end, a total of 164 patients (82 men and 82 women) were successfully enrolled in the study.

The participants had a median age of 53.68 years, with an interquartile range (IQR) of 12.51. The mean duration of T2DM was 9.3 years (IQR 5.3). The mean body mass index (BMI) was calculated to be 29.39 kg/m^2^ (IQR 6.67). A total of 35 patients (21.34%; 18 men and 17 women) had a normal body weight (typically defined for the Polish population as BMI <25.0 kg/m^2^), 50 patients (30.49%; 22 men and 28 women) were overweight (BMI 25.0–29.9 kg/m^2^), and 79 participants (48.17%; 42 men and 37 women) were classified as obese (BMI ≥30.0 kg/m^2^)—this group will be referred to as “diabesity” patients throughout the article. Among the patients with diabesity, there were 51 patients with first-degree obesity (with BMI 30.0–34.9 kg/m^2^; 64.55%: 24 men and 27 women), 20 patients with second-degree obesity (with BMI 35.0–39.9 kg/m^2^; 25.32%: 13 men and 7 women), and 8 patients with third-degree obesity (with BMI 40.0 kg/m^2^ and above; 10.13%: 5 men and 3 women). There was no statistically significant difference seen in terms of gender (*p* = 0.587), age (*p* = 0.196), and duration of diabetes (*p* = 0.211) among the groups that were classified based on body weight criteria. A total of 99 patients (60.36%) were diagnosed with well-controlled metabolic diabetes (defined according to the guidelines of the Polish Diabetes Association, based on the patient’s age and clinical parameters [15]).

### 2.2. Anthropometrical, Biochemical, and Liver Stiffness Analyses

Weight and height were measured to calculate BMI (in kg/m^2^). Biological material (blood and serum) was collected for laboratory analyses encompassing various (especially metabolic and hepatic) parameters: peripheral blood morphology (with: WBC—white blood cells; RBC—red blood cells; Hb—hemoglobin; Hct—hematocrit; MCV—mean corpuscle volume; MCH—mean corpuscular hemoglobin; MCHC—mean corpuscular hemoglobin concentration; PLT—platelets; RDW—red cell distribution width; PDW—platelets distribution width; MPV—mean platelet volume; P-LCR—platelet-large cell ratio; PCT—plateletcrit), ALT (alanine aminotransaminase), AST (asparagine aminotransaminase), GGTP (gamma-glutamyl transferase), total bilirubin, albumin, glucose, insulin (with estimation of HOMA-IR index), HbA1c, lipid profile (total cholesterol, HDL-C—high density lipoprotein cholesterol, LDL-C—low density lipoprotein cholesterol, triglycerides), INR (international normalized ratio), and prothrombin time.

Every patient underwent an elastography liver examination using the FibroScan device (model 530 compact with both probes: M and XL; Echosens, France), conducted by a single, experienced, and authorized operator (registration: XL22900072245), to evaluate liver fibrosis in kPa (this diagnosis was established at values >7 kPa)—device software incorporates an embedded automatic probe selection tool that provides recommendations for the most suitable probe based on a real-time assessment of the skin-to-liver capsule distance for each individual patient.

### 2.3. Statistical Analysis

The acquired findings were subjected to statistical analysis utilizing Microsoft Office Excel 365 and STATISTICA 13.0 PL. Due to the fact that most quantitative variables were distributed abnormally, the median and interquartile range (IQR) were used to describe central tendencies, and non-parametric coefficients and tests (Mann–Whitney U-test, Kruskal–Wallis H-test, and Spearman’s correlation coefficient) were used for the analyses. Furthermore, the χ^2^ test and logistic regression analysis were also conducted. A significance level of α = 0.05 was adopted for the entire statistical analysis.

## 3. Results

**Figure 1:**
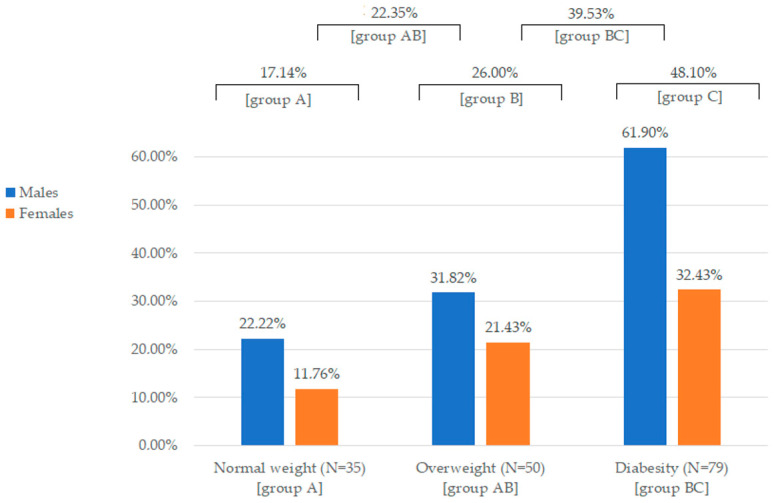
The prevalence of liver fibrosis in relation to gender and body weight attributes.

Liver fibrosis, as determined by FibroScan-determined transient elastography, was found in 57 patients with T2DM (34.76%), being more prevalent in men than women (45.12% vs. 24.39%, *p* = 0.011). According to the data presented in Figure 1, liver fibrosis occurred significantly more often among overweight and diabesity patients compared to those with normal body weight (*p* = 0.002).

The relative risk of liver fibrosis in diabetes turned out to be significantly higher in the group of males (RR 1.85; 95%CI: 1.18–2.90) and patients with diabesity (RR 2.81; 95%CI: 1.31–6.02; *p* = 0.008). When considering individuals with diabesity, higher degrees of obesity are correlated with an increased risk of liver fibrosis: in first-degree cases, the risk was 2.4 times higher (RR 2.40; 95%CI: 1.08–5.34; *p* = 0.032), in second-degree cases, it was 3.5 times higher (RR 3.50; 95%CI: 1.55–7.88; *p* = 0.003), and in the third-degree obesity, risk of hepatofibrosis was 3.65 times higher (RR 3.65; 95%CI: 1.48–9.01; *p* = 0.005). On the other hand, patients with well-controlled metabolic diabetes, as assessed by the percentage of HbA1c, had a 55% lower risk (RR 0.45; 95% CI: 0.30–0.68) of liver fibrosis.

Table 1 displays the attributes and comparison of parameters for all patients in respect to the presence or absence of liver fibrosis. Patients afflicted with liver fibrosis exhibited the following characteristics in contrast to those who did not have fibrosis: higher BMI, greater RBC with lover MCV and anisocytosis (RDW-CV), lower PLT, higher levels of all aminotransferases (ALT, AST, GGTP), lower albumin concentration, higher fasting blood glucose, higher percentage of glycated hemoglobin, lower HDL-C, and higher trigliceryde concentration. Table 2 illustrates disparities among people who have both type 2 diabetes and obesity (diabesity). In these patients, liver fibrosis is associated with lower MCV, lower platelet count, anisocytosis (indicated by the red cell distribution width coefficient of variation), lower plateletcrit, higher aminotransferase activity, lower albumin concentration, higher percentage of HbA1c, surprisingly better lipid parameters (except triglicerydes), and with marginally lower white and higher red blood cells counts. Patients diagnosed with diabesity had a higher risk of developing liver fibrosis when their levels of certain liver enzymes were elevated: ALT (RR 2.47; 95%CI 1.67–3.66), AST (RR 2.07; 95%CI 1.34–3.22), and GGTP (RR 1.78; 95%CI 1.14–2.79). Furthermore, there was an increased relative risk of liver fibrosis in the presence of anisocytosis (RR 2.43; 95%CI: 1.61–3.68), as well as in cases of thrombocytopenia with RR 2.14; 95%CI: 1.64–2.81.

The hepatic elastographic stiffness index (indicating the severity of liver fibrosis, measured in kPa) in patients with T2DM was significantly correlated with several factors: BMI (R = 0.222; *p* = 0.008), MCV (R = −0.196, *p* = 0.020), RDW-CV (R = 0.211; *p* = 0.013), ALT (R = 0.336; *p* < 0.001), AST (R = 0.259; *p* = 0.002), GGTP (R = 0.418; *p* < 0.001), albumin (R = −0.185; *p* = 0.028), HDL-C (R = −0.183; *p* = 0.031), fasting glucose (R = 0.274; *p* = 0.001), HbA1c (R = 0.297; *p* = 0.005)—there were also borderline significant correlations for PLT (R = −0.161, *p* = 0.058) and triglycerides (R = 0.165, *p* = 0.053); with the exception of the correlation pertaining to GGTP, all other correlations can be categorized as weak correlations based on the Chaddock scale. Correlations exclusively evaluated for individuals diagnosed with diabesity are presented in Table 3.

**Figure 2:**
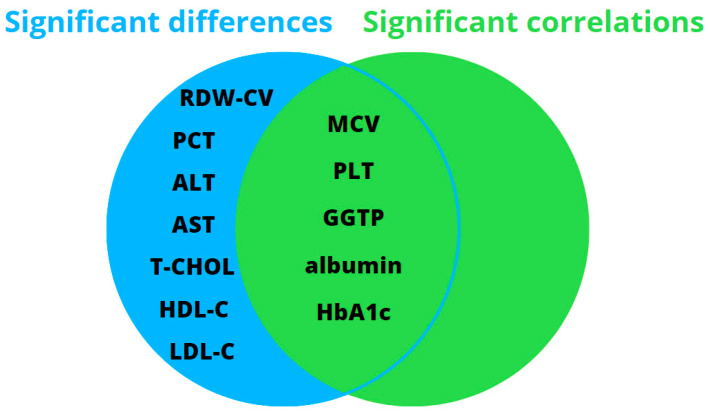
Scheme of crucial parameters in the group of patients with diabesity.

When looking at a group of diabesity patients for statistically significant differences (*p*-value of the Mann–Whitney U-test <0.05) in different laboratory parameters (based on the presence or absence of liver fibrosis) and the correlations of these parameters with liver stiffness, the key parameters that emerged as significant and common factors were mean corpuscular volume, platelet count, GGTP activity, albumin concentration, and the percentage of glycated hemoglobin, as illustrated in Figure 2.

The regression analysis revealed significant and separate connections between liver stiffness and albumin concentration (β = −0.391, SE = 0.159, *p* = 0.021), as well as between liver stiffness and GGTP activity (β = 0.358, SE = 0.161, *p* = 0.033). The relationships for other parameters did not reach statistical significance. These characteristics can be considered essential for effectively managing hepatofibrosis in diabetic-obese individuals.

Curiously, none of the patients we assessed who exhibited signs of hepatofibrosis were previously aware of these indicators. Furthermore, during the medical interview, they explicitly stated that they had never been informed about the necessity of undergoing examinations in this specific area during previous visits to other healthcare providers.

## 4. Discussion

Until now, surprisingly, the issue of hepatofibrosis in patients with concurrent obesity and diabetes was not extensively explored in the scientific literature, in contrast to the well-documented topic of hepatosteatosis. Michaelidou et al.’s review on diabesity suggests that MAFLD may be an initial result of T2DM and metabolic syndrome, impacting the end-organs [16]. Hepatosteatosis is the primary cause of MAFLD; however, it is worth mentioning that the advancement of this condition is also affected by its tendency to develop into hepatofibrosis. Thus, it is essential to give proper attention to this matter.

The concept of “diabesity” (initiated by Ethan A. H. Sims from the University of Vermont College of Medicine [17]) in the context of liver fibrosis appeared in the study by Arnaiz et al., presented as an electronic poster at the 22nd European Congress of Endocrinology in 2020. This study included 95 obese people, 23 of whom had diabetes—they were more susceptible to advanced hepatofibrosis, with prevalence rates ranging from 17.4% to 18.4% compared to 1.4% to 2.8% in non-diabetics. This investigation used widely known non-invasive serum scales: the NAFLD Fibrosis Score, Hepamet, and FIB-4; elastographic tests and other methods were not used [18]. Non-invasive markers for liver fibrosis assessment include low specificity, many false-positive results, difficulty distinguishing fibrosis levels, and many inconclusive results [19]. According to our best knowledge, our study represents a pioneering effort in addressing the topic of hepatofibrosis in individuals with coexisting diabetes and obesity (including those in an intermediate state, such as overweight). Notably, the investigation explores the associations between various laboratory indicators and liver stiffness across distinct patient cohorts.

As mentioned, in clinical practice, many scales are utilized to predict liver fibrosis non-invasively: AST to platelet ratio index, age-platelet index, European Liver Fibrosis score, Fibrometer, Hepascore, FIB-4, SteatoTest, NAFLD fibrosis score, cirrhosis discriminate score, BARD score, Hui model, FibroMeter NAFLD, Fibrosis Probability Index, Lok index, Fibro Q, and others [20]. Several models and equations incorporate indicators such as BMI and the presence or absence of diabetes. These markers include the BARD score [21] and NAFLD fibrosis score [22]. Additionally, the calculation of the Fibrometer value necessitates the determination of fasting glucose and body weight [23]. The sensitivity, specificity, diagnostic accuracy, and precision of these markers exhibit variability across different clinical scenarios in which they are evaluated. However, the presence of these markers signifies that the connection between obesity and diabetes in the context of hepatofibrosis etiopathogenesis has already been acknowledged earlier.

We found liver fibrosis in 34.76% of patients with T2DM—in the diabesity group, the prevalence of liver fibrosis was considerably higher at 61.90%. The results match earlier studies in this sector, supporting past understanding. Lomonaco et al. found liver fibrosis in 21% of 561 patients with T2DM using elastography [24], and Sporea et al. observed 19.6% of T2DM patients had advanced fibrosis and 8.2% had cirrhosis in Romania using the same method [25]. Patients with diabetes and liver fibrosis, depending on stage, can be obese in up to 87.2% of cases, according to Ciardullo et al. [26]. Barb et al. found a 1.8–2.5-fold increase in liver fibrosis risk with diabetes and obesity; however, instead of measuring liver stiffness, the authors used the non-invasive risk indicator FIB-4 for hepatofibrosis (but the study included 1574 obese people without diabetes and 571 with diabetes and obesity [27]).

The finding by Gupta et al. is noteworthy among studies on hepatic stiffness. Researchers found a strong correlation between hepatic stiffness and HbA1c (R = 0.14; *p* < 0.05) and GGTP (R = 0.26; *p* < 0.01) in diabetic patients [28]. When evaluating correlations only within the diabetic group, Gupta et al.’s findings are mostly like ours. The average BMI of the study group was 31 kg/m^2^, which may explain this uniformity. This information is important since Mangla et al. found a favorable connection between liver stiffness and cardiovascular events. Thus, such events must be prevented [29]. Dwinata and Gracen found that patients using sodium-glucose co-transporter 2 inhibitors (SGLT2i) or glucagon-like peptide-1 receptor agonists (GLP1Ra) have less incidences of diagnosed liver fibrosis [30,31]. While originally developed for diabetes treatment, these drugs have garnered attention from medical professionals specializing in cardiology due to their potential therapeutic effects in mitigating liver fibrosis.

In our study, patients with diabesity and liver fibrosis were characterized by lower mean corpuscular volume (MCV), lower platelet count, higher RDW-CV, higher aminotransferase activity, lower albumin levels, and a higher percentage of HbA1c. The key variables that establish a connection between characteristics and correlations were identified as MCV, PLT, GGTP activity, albumin concentration, and HbA1c; however, regression analysis revealed statistically significant and independent correlations between liver fibrosis and only albumin concentration or GGTP activity.

Results concerning erythrocytes are particularly interesting. In 2013, a Seoul study of 24,575 NAFLD patients found that increased RDW independently predicted progressive fibrosis (assessed by BARD and FIB-4 indices) [32]. Similar results were also achieved by Turkish and Chinese research groups [33,34]. Many other studies have highlighted the RDW parameter’s role in predicting liver fibrosis and cirrhosis, although usually in the context of viral or autoimmune liver inflammation [35,36,37,38,39,40,41]. There is a hypothesis that persistent inflammation and oxidative stress may cause irregular erythrocytes, which increase red cell distribution width [42]. Lower MCV and anisocytosis in our patients with hepatofibrosis in the context of diabesity could also suggest disturbances in iron metabolism. Nevertheless, our protocol did not include comprehensive investigations pertaining to iron metabolism, such as iron, ferritin, and transferrin. Several published studies have highlighted the significance of iron in the development of advanced liver fibrosis [43,44]. This may be due to the complex systemic regulation of iron levels by the liver hormone hepcidin—in NAFLD, iron deficiency may reduce hepcidin [45,46]. In contrast, HFE gene variants (particularly C282Y and H63D) associated with NAFLD increased liver iron levels and decreased serum iron [47,48]. Additionally, starvation, bone marrow suppression, thyroid dysfunction, vitamin B12 insufficiency, and cardiovascular diseases can cause anisocytosis and microcytosis [42]. This work provides a solid foundation for future research on whether optimum iron metabolism can slow liver fibrosis.

The platelet count is a widely studied parameter that is commonly used both independently and as part of various markers (such as APRI, FIB-4, NAFLD score, Forns index, Fibrometer, Lok index, GUCI, CDS, King’s score, Pohl index, and VITRO score) for the non-invasive evaluation of the risk of liver fibrosis [49]. Ho’s study found that numerous tumors, hepatitis C infection, and low platelet counts are the greatest independent risk factors for severe liver fibrosis [50]. The cause of a reduced platelet count in liver fibrosis, especially cirrhosis, is multifaceted. Several factors may cause this condition, including platelet sequestration in the spleen, platelet inhibition in the bone marrow, reduced liver thrombopoietin production, and platelet destruction through autoimmune mechanisms. Blood platelets play a major role in liver inflammation through their interactions—these interactions cause liver healing, regeneration, and necroinflammation. This process may start early in NAFLD or hepatitis C-induced liver disorders [51,52,53,54,55]. Based on the Chaddock scale, for our patients with both diabetes and obesity, the correlation between platelet count and liver stiffness was relatively weak, albeit statistically significant (R = −0.302, *p* = 0.016). Without prior verification of such a properly curated cohort from this perspective, it is difficult to make definitive references to the literature. Further research should determine how much platelet deficiency (or elevation) affects diabesity patients.

BMI, AST, and GGTP were independent liver fibrosis risk factors, according to Ciardullo et al. [26]. In our study among patients with diabesity, the correlation between liver stiffness index and GGTP activity was the strongest (R = 0.409). The role of this parameter has been appreciated by many researchers. For example, gamma-glutamyl transferase peptide is a component of well-validated non-invasive liver fibrosis markers such as Forns score [56], Hepascore [57], and STEATO test panel [58], but only the latter was designed for metabolic disorders and NAFLD, the others for hepatitis C. Researchers found that the ratio of GGTP to HDL-C concentrations can be a biomarker for NAFLD or MAFLD, especially in obese patients [59,60,61,62]. Our study, similar to the study by Ciardullo et al., is important evidence that GGTP activity should be considered in diabesity hepatic fibrosis.

Albumin concentration is essential for liver function assessment. In severe liver fibrosis and cirrhosis, albumin synthesis is hindered, lowering albumin concentration [63]. Tada et al. found that albumin concentration is associated with liver fibrosis progression in T2DM, particularly middle-aged patients [64]. Albumin is found in the HUI model, SHASTA index, and NAFLD fibrosis score—only the last of the markers has been verified for usage in NAFLD patients (the other two are suited for hepatotropic viral infections) [22,65,66]. The study recently published in the Journal of Controlled Release suggests the utilization of an albumin-fused long-acting fibroblast growth factor 21 (FGF21) analogue as a potential treatment for NAFLD, especially in obese patients who are at risk of hepatofibrosis [67]. The expression of FGF21 is positively correlated with liver triglycerides and liver fat metabolism—high FGF21 levels are found in obesity, T2DM, and metabolic syndrome [68,69,70]. A study on an animal model tested whether nanoparticles of berberine and bovine albumin may treat liver fibrosis—the mouse model showed efficacy, but these findings cannot be directly applied to human therapy (particularly since this study addressed liver damage caused by chemical means like CCl_4_ rather than metabolic diseases) [71]. The role of impairments in albumin function in the pathogenesis of liver disease is currently uncertain, and it is not yet clear whether these impairments are a cause or a result of the disease. This aspect needs further study to determine a causal link and explore therapeutic options.

Glycated hemoglobin is essential for diagnosing, monitoring metabolic control, and evaluating diabetes treatment. Additionally, it is employed in instances of prediabetic conditions. Our study found that people who follow Polish Diabetes Association standards for metabolic diabetes control had a twofold decreased incidence of hepatofibrosis. Multiple studies have consistently demonstrated a strong association between hemoglobin A1c and the likelihood of developing liver fibrosis, even among people without carbohydrate metabolic issues. Chen et al. found that HbA1c may contribute to non-alcoholic fatty liver disease even at normal levels. The values of our patients, both in the general study population and among those with diabesity, deviated from normative ranges, but given the patho-physiological factors involved, such as nitric oxide, hypoxia, and the receptor for advanced glycation end products (RAGE), such deviations are not unusual [72]. Tanaka et al. discovered that individuals with a HbA1c ≥6.5% had a 170% higher likelihood of developing hepatofibrosis (assessed by FIB-4 test results) in a study of 6927 NAFLD patients [73]. In a study involving 774 diabetes patients in New York, researchers found that HbA1c is the only and strongest indicator of liver fibrosis based on elastographic examination (β = 0.37; 95%CI 0.04–0.69)—the HOMA-IR index did not show a similar relationship, similarly to our study [74]. In the cross-sectional Gutenberg Health study of 14,950 participants, increased HbA1c was connected to an 184% higher risk of hepatofibrosis (conducted through non-invasive serum markers such as NFS, FIB-4, and APRI) [75]. In contrast, Ciardullo and Perseghin discovered in 744 patients with T2DM (with the utilization of vibration-controlled transient elastography) that there is no statistically significant difference in HbA1c levels between those with advanced hepatofibrosis (>9.7 kPa) and those without this condition—not like in prior research [76]. Patient education on the importance of healthy glycemic levels may significantly influence the treatment of diabetes and related disorders, such as obesity and cardiovascular disease.

In a study conducted at Huazhong University of Science and Technology in Wuhan, reduced HDL-C concentration was linked to a nearly fivefold higher risk of advanced fibrosis (defined as an NFS score >0.676)—no other lipid parameters showed statistically significant differences or correlations [77]. Atypical observation is that our patients with hepatofibrosis also exhibited potentially better lipid parameters; however, it is essential to note the wide interquartile range of these parameters, which can lead to apparent statistical significance. Due to the lack of prior observations, it is important to interpret this finding cautiously and scrutinize it in future studies, such as the upcoming follow-up study with a larger patient cohort.

The role of diet is crucial in the cause and progression of hepatofibrosis, particularly in individuals with type 2 diabetes mellitus (T2DM). For example, excessive amount of cholesterol in the diet has a negative impact on hepatocytes, Kupffer cells, hepatic stellate cells, cholangiocytes, and liver sinusoidal endothelial cells through the formation of intraplasmic crystals [78]. In contrast, a high-fiber diet lowers proinflammatory substances like tumor necrosis factor-alpha, interleukin 1, and interleukin 6 and increases IL10 or interferon gamma, which reduces liver injury [79]. People with type 2 diabetes and obesity often have poor dietary habits. Doctors and other medical professionals should emphasize this during patient interactions. In this pilot study, we did not analyze patient diet diversity; we wish to research this further in the future.

An alarming and disheartening revelation is that liver fibrosis patients reported a paucity of guidance on the importance of regular liver health checks. This discovery is alarming because NAFLD is a key component of the metabolic syndrome, which increases the risk of cardiovascular disease. Carrieri et al. stressed that accurate liver disease progression knowledge affects lifestyle changes [80]. These findings should prompt a patient- and professional-focused educational campaign to raise liver health awareness. The MAFLD e-academy for medical postgraduate education made a strong start in Poland.

It is crucial to highlight that diabetes is closely related to the metabolic syndrome, regardless of the specific definition used (such as the IDF from 2005, IDF from 2009, or the consensus of Polish scientific societies from 2022 [81,82,83]). Diabetes and abdominal obesity are probably two key metabolic syndrome diagnostic criteria. Should patients with diabesity be considered “pre-metabolic syndrome” patients? A definitive solution is difficult without more investigation; however, undeniably, each component of the metabolic syndrome increases cardiovascular risk, and when combined, the risk is substantially higher.

While not the main focus of the study, 34 out of 79 patients with diabetes (including all grade III obese patients) expressed a willingness to intensify their treatment after receiving the elastography results and understanding the concept of MAFLD, which increases cardiovascular risk. This treatment intensification may include GLP1Ra medication (which is expensive and in high demand among overweight and obese patients in Poland) or bariatric surgery. According to current knowledge, the main goal of treating NAFLD with fibrosis or cirrhosis is weight loss of 7–10% through lifestyle changes, medication, and metabolic surgery [84,85,86]. Several studies have also suggested the utilization of pioglitazone [87,88] or vitamin E (although the latter is primarily justified for individuals without diabetes) [89]. The planned study follow-up will validate our patients’ declarations’ final effect.

In every study, researchers should know their limits; this manuscript is no exception. Firstly, despite achieving minimum sample size standards, this study had a small participant pool due to financial and temporal constraints (like the Fibroscan device’s limited availability); the results need further validation for this reason. Secondly, the study did not consider many factors that could affect liver health, such as a thorough assessment of the patients’ eating patterns and physical exertion. Thirdly, the diagnosis of obesity in our group was determined solely by calculating the body mass index; waist circumference was not considered due to missing data, potentially leading to the exclusion of patients with a BMI <30 kg/m^2^ but with a waist circumference exceeding 80/94 cm or 88/102 cm, depending on the specific criteria used. An interesting complement to the study may be evaluating diabesity patients’ genetic profiles—as we know, genetic variations and mutations can affect liver damage susceptibility, such as INFL genotypes [90]. We used elastographic examination data to diagnose liver fibrosis in our investigation, which raised concerns because liver biopsy is still the gold standard for the diagnosis of hepatofibrosis. However, all patients with liver fibrosis, especially those with severe fibrosis, were sent to a specialized hepatology clinic for appraisal and treatment—in our group of patients, diagnosis was never questioned, and the results seem to validate the Fibroscan device’s great sensitivity in diagnosing advanced liver fibrosis [91]. Taking into account the above issues and drawbacks, the results of this study should be considered preliminary—we plan to expand the investigation in the near future.

Notwithstanding these constraints, it is important to reiterate that the current investigation stands out as one of the few studies that centers its attention on a cohort of patients who exhibit both diabetes and obesity simultaneously. Given the notable epidemiology of both aforementioned medical conditions, it appears imperative to enhance the breadth and quantity of research pertaining to these subjects in forthcoming investigations. We believe that this manuscript will serve as inspiration for other researchers in this regard—for the researchers involved in this study, the current findings also serve as a preliminary exploration that paves the way for future investigations.

## 5. Conclusions

People with type 2 diabetes have a higher risk of developing hepatofibrosis, especially if they are classified as having diabesity. An association was observed between liver stiffness and various hematological and biochemical markers in patients with both diabetes and obesity. Albumin concentration and GGTP activity are essential for the development of effective diagnostic and treatment solutions for these individuals. Additionally, mean corpuscular volume, platelet counts, and hemoglobin A1c percentage are also important. In routine clinical practice, physicians treating patients with metabolic problems (such as diabetes and obesity) should closely monitor the above-mentioned parameters to promptly identify the necessity of evaluating the liver for fibrosis.

This study one of the few that focuses on a specific patient population, specifically patients who have both diabetes and obesity simultaneously. Further inquiry is required to comprehensively examine hepatic fibrosis in patients with diabetes, especially when accompanied by weight-related comorbidities such as overweight and obesity.

## Figures and Tables

**Table 1:** Characteristics of patients’ parameters, depending on the presence or absence of hepatofibrosis (diabetes patients).

Parameter	Median (IQR)—Diabetes Patients			Mann– Whitney U-Test *p*-Value (with vs. without Liver Fibrosis)
	All Patients (*n* = 164)	Patients with Liver Fibrosis (*n* = 57)	Patients without Liver Fibrosis (*n* = 107)	
Age [years]	53.68 (12.51)	53.22 (8.23)	54.07 (13.74)	0.748
BMI [kg/m^2^]	29.39 (6.67)	31.24 (6.75)	28.19 (6.49)	0.001
WBC [×10^3^/μL]	7.11 (2.42)	7.00 (2.77)	7.24 (2.27)	0.572
RBC [×10^6^/μL]	4.67 (0.64)	4.67 (0.61)	4.59 (0.61)	0.014
Hb [g/dL]	13.70 (1.90)	14.40 (3.30)	13.70 (1.10)	0.401
Hct [%]	41.90 (4.20)	41.70 (8.80)	41.65 (3.80)	0.464
MCV [fl]	91.10 (5.20)	89.30 (5.20)	92.20 (4.00)	<0.001
MCH [pg]	30.50 (2.70)	30.20 (3.10)	30.50 (2.10)	0.126
MCHC [g/dL]	33.30 (1.30)	33.50 (2.10)	33.10 (1.10)	0.945
PLT [×10^3^/μL]	248.50 (99.00)	223.00 (86.00)	254.00 (101.00)	0.024
RDW-SD [fl]	44.65 (4.40)	45.30 (7.00)	44.40 (4.00)	0.224
RDW-CV [%]	13.40 (1.35)	13.90 (1.80)	13.30 (0.90)	<0.001
PDW [fl]	12.30 (2.90)	13.00 (3.40)	12.25 (2.60)	0.077
MPV [fl]	10.40 (1.00)	10.40 (1.50)	10.35 (1.20)	0.140
P-LCR [%]	28.90 (9.50)	29.00 (13.30)	28.30 (9.40)	0.126
PCT [%]	0.25 (0.08)	0.24 (0.09)	0.26 (0.11)	0.053
ALT [U/L]	26.50 (18.10)	36.60 (25.00)	23.65 (12.50)	<0.001
AST [U/L]	23.70 (14.10)	30.40 (14.10)	22.80 (10.70)	<0.001
GGTP [U/L]	23.85 (23.10)	29.80 (57.50)	22.40 (13.00)	0.002
Albumin [g/L]	42.20 (3.50)	40.40 (3.60)	42.90 (2.50)	0.001
Fasting glucose [mg/dL]	118.90 (60.80)	121.60 (102.10)	109.30 (34.60)	0.002
Insulin [mU/L]	10.99 (12.25)	14.45 (11.86)	10.21 (8.74)	0.136
HOMA-IR	3.39 (4.26)	4.95 (6.72)	2.98 (3.07)	0.069
HbA1c [%]	6.30 (1.70)	6.70 (1.90)	6.20 (1.30)	<0.001
Total cholesterol [mg/dL]	188.80 (74.70)	177.20 (80.80)	188.25 (74.00)	0.681
HDL-C [mg/dL]	50.20 (19.05)	49.30 (23.30)	52.00 (18.90)	0.032
LDL-C [mg/dL]	101.40 (57.80)	86.50 (61.60)	105.90 (58.40)	0.982
Triglicerydes [mg/dL]	106.05 (64.30)	110.90 (89.70)	100.35 (59.80)	0.067
INR	1.03 (0.12)	1.04 (0.16)	1.03 (0.11)	0.499
Prothrombin time [s]	11.60 (1.20)	11.80 (1.60)	11.60 (1.20)	0.297

**Table 2:** Characteristics of patients’ parameters, depending on the presence or absence of hepatofibrosis (diabesity patients).

Parameter	Median (IQR)—Diabesity Patients (*n* = 79)		*p*-Value (Mann– Whitney U-Test)
	Patients with Liver Fibrosis (*n* = 38)	Patients without Liver Fibrosis (*n* = 41)	
Age [years]	53.23 (6.72)	50.62 (14.37)	0.192
BMI [kg/m^2^]	33.80 (5.65)	32.91 (3.48)	0.368
WBC [×10^3^/μL]	7.00 (2.19)	7.89 (1.73)	0.097
RBC [×10^6^/μL]	4.86 (0.66)	4.71 (0.46)	0.073
Hb [g/dL]	14.65 (2.60)	14.00 (1.10)	0.153
Hct [%]	43.25 (7.60)	42.50 (1.90)	0.320
MCV [fl]	89.30 (5.10)	92.50 (3.40)	<0.001
MCH [pg]	30.10 (2.85)	30.40 (1.90)	0.352
MCHC [g/dL]	33.50 (2.05)	33.10 (1.00)	0.559
PLT [×10^3^/μL]	243.50 (75.00)	292.00 (106.00)	0.004
RDW-SD [fl]	44.35 (7.70)	44.40 (2.10)	0290
RDW-CV [%]	14.10 (2.20)	13.40 (0.90)	0.006
PDW [fl]	13.50 (3.95)	12.40 (2.50)	0.165
MPV [fl]	10.60 (1.65)	10.45 (1.05)	0.186
P-LCR [%]	30.80 (14.95)	29.10 (9.60)	0.190
PCT [%]	0.25 (0.09)	0.29 (0.12)	0.038
ALT [U/L]	34.00 (25.00)	22.50 (9.20)	0.007
AST [U/L]	29.10 (14.10)	22.60 (7.30)	0.001
GGTP U/L]	34.40 (34.85)	25.60 (7.10)	0.021
Albumin [g/L]	40.95 (2.50)	43.20 (2.20)	0.006
Fasting glucose [mg/dL]	140.80 (107.40)	126.00 (48.50)	0.165
Insulin [mU/L]	14.85 (9.44)	10.56 (9.92)	0.352
HOMA-IR	5.08 (6.49)	3.40 (3.11)	0.192
HbA1c [%]	7.50 (2.30)	6.35 (0.90)	0.018
Total cholesterol [mg/dL]	176.60 (75.00)	208.00 (35.10)	0.017
HDL-C [mg/dL]	50.50 (20.85)	50.00 (16.60)	0.032
LDL-C [mg/dL]	85.70 (56.60)	119.20 (24.50)	0.010
Triglicerydes [mg/dL]	110.55 (145.90)	120.15 (92.95)	0.973
INR	1.06 (0.14)	1.04 (0.10)	0.338
Prothrombin time [s]	12.00 (1.40)	11.70 (1.10)	0.141

**Table 3:** Correlation with liver stiffness elastographic index and other parameters in diabesity.

Parameter	R-Spearman	*p*-Value
Age [years]	0.089	0.439
BMI [kg/m^2^]	0.063	0.632
WBC [×10^3^/μL]	−0.182	0.158
RBC [×10^6^/μL]	0.229	0.068
Hb [g/dL]	0.236	0.061
Hct [%]	0.107	0.401
MCV [fl]	−0.249	0.047
MCH [pg]	0.026	0.839
MCHC [g/dL]	0.205	0.104
PLT [×10^3^/μL]	−0.302	0.016
RDW-SD [fl]	−0.011	0.934
RDW-CV [%]	0.135	0.288
PDW [fl]	0.059	0.646
MPV [fl]	0.022	0.866
P-LCR [%]	0.013	0.918
PCT [%]	−0.239	0.061
ALT [U/L]	0.216	0.092
AST [U/L]	0.247	0.056
GGTP U/L]	0.409	<0.001
Albumin [g/L]	−0.321	0.011
Fasting glucose [mg/dL]	0.248	0.052
Insulin [mU/L]	0.079	0.547
HOMA-IR	0.179	0.174
HbA1c [%]	0.327	0.012
Total cholesterol [mg/dL]	−0.203	0.108
HDL-C [mg/dL]	−0.229	0.070
LDL-C [mg/dL]	−0.214	0.089
Triglicerydes [mg/dL]	0.055	0.673
INR	0.166	0.208
Prothrombin time [s]	0.166	0.208

## Data Availability

The data can be made available upon reasonable request—please contact the correspondence author. The data are not publicly available due to fact, that containing information that could compromise the privacy of research participants.
